# P02.116. The relationship between deqi and the effect of acupuncture

**DOI:** 10.1186/1472-6882-12-S1-P172

**Published:** 2012-06-12

**Authors:** Y Choi, S Cho, J Lee, W Moon, D Yoo

**Affiliations:** 1 Hospital of Korean Medicine, Kyung Hee University Medical Center, Seoul, Republic of Korea

## Purpose

There is an experimental study that suggests deep needling with rotation produces higher acupuncture needling sensation than superficial needling with mock rotation. Also, there are opposing results about the relationship between acupuncture needling sensation and analgesic effect. In this study, we intend to investigate the relationship between acupuncture needling sensation and analgesic effect according to acupuncture stimulation.

## Methods

Fifty healthy volunteers received 3 different forms of acupuncture in a single-blinded crossover design; these included superficial needling (0.3 cm), deep needling (2 cm), and needling with bi-directional rotation. The time between the interventions was more than 48 hours. All forms of acupuncture were applied unilaterally on the left leg at standard acupuncture points: SP 6, SP 9, ST 36, and GB 39. The effects of acupuncture were evaluated using pressure pain threshold. Each participant completed the Subjective Acupuncture Sensation Scale (SASS).

## Results

Both SASS and increases in pressure pain threshold were largest in needling with rotation followed by deep needling and superficial needling. An ANOVA analysis was carried out in order to see whether there is a significant difference; both had p values lower than 0.001. Also, the correlation between the sum of SASS and changes in pressure pain threshold was calculated using Spearman’s rho; there was a significant correlation (p=0.027).

## Conclusion

Acupuncture needling sensation and pressure pain threshold increased according to depth and rotation of acupuncture. There is a significant correlation between acupuncture needling sensation and increases in pressure pain threshold. It seems that deqi plays an important role in revealing the effect of acupuncture.

